# Shelter preference and behavior of dairy cows managed outdoors during calving in temperate winter conditions

**DOI:** 10.3168/jdsc.2023-0485

**Published:** 2024-02-01

**Authors:** Fabiola Matamala, Inès de Freslon, Maria José Hötzel, Pilar Sepúlveda-Varas

**Affiliations:** 1 Escuela de Graduados, Facultad de Ciencias Veterinarias, Universidad Austral de Chile, Valdivia, Chile, 5090000; 2 Comité Institucional de Uso y Cuidado Animal, Universidad Austral de Chile, Valdivia, Chile, 5090000; 3 Laboratório de Etologia Aplicada e Bem-Estar Animal, Departamento de Zootecnia e Desenvolvimento Rural, Universidade Federal de Santa Catarina, Florianópolis, Brazil, 88034-001; 4 Instituto de Ciencias Clínicas Veterinarias, Facultad de Ciencias Veterinarias, Universidad Austral de Chile, Valdivia, Chile, 5090000; 5 Programa de Bienestar Animal, Universidad Austral de Chile, Valdivia, Chile, 5090000

## Abstract

This study aimed to assess the preference and use of an artificial shelter in dairy cows managed outdoors at calving during winter. We also evaluated whether this preference would be influenced by weather conditions (rain, air temperature, or wind) or the time of the day at calving. Two weeks before their expected calving date, 18 cows were paired based on body weight, body condition score, parity, and expected calving date. Each pair was assigned to an open paddock (42 m^2^/cow) with a bare soil surface, high mud content, and access to an artificial shelter until calving. The shelter consisted of a 6 × 3 m metal structure, with 3 sides covered with zinc sheets, a polycarbonate roof, and a thick layer of dry sawdust covering the ground surface. Shelter use and cow behavior inside it (lying and sleeping) were continuously recorded via video and analyzed using continuous observation. Observations were divided into 2 periods: the day before calving (24 to 48 h before calving) and the day of calving (24 h before calving until calving). Ninety-four percent (15/16) of the cows preferred to calve inside the artificial shelter, and this preference was not affected by the time of day or weather conditions. Regardless of the day of study (the day before calving or the day of calving), cows spent approximately 64% of their daily time inside the shelter, and once inside, they spent most of the time lying down. Our findings indicate dairy cows prefer using an artificial shelter as a calving and lying place, suggesting that opportunities for protection should be provided when they are managed outdoors in muddy paddock conditions.

## Highlights

• Most cows preferred to use the artificial shelters to calve.
• Cows spent more than 60% of the day inside the artificial shelter.
• Cows used the artificial shelter mainly to lie down.

## Graphical Abstract

**Figure:**
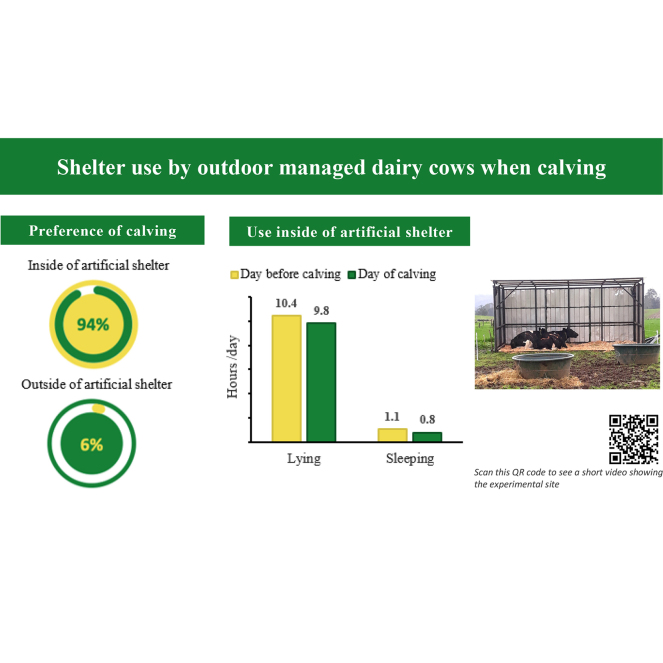
**Summary** In pasture-based systems in temperate regions, dairy cows frequently calve exposed to inclement winter weather and muddy conditions underfoot, but it remains unclear whether cows prefer access to shelter at calving and the factors that affect their preferences. We assessed the preference and use of an artificial shelter for cows close to calving. Most cows preferred calving inside the artificial shelter, regardless of weather conditions (rain, temperature, humidity, or wind) or whether it was night or daytime. Cows also spent a considerable portion of the day inside the artificial shelter, using it mainly for lying, both on the day before calving and the day of calving. This study shows evidence that outdoor-managed dairy cows use the shelter to seek a dry surface as calving approaches.

Late winter or early spring outdoor calving is a common practice among farmers in some temperate regions with pasture-based systems (e.g., southern Chile and New Zealand), where peri-parturient dairy cows are temporarily relocated to designated areas or “sacrifice” paddocks during rainy periods. This practice aims to safeguard the soil from damage caused by trampling and prevent subsequent reductions in pasture growth. Late pregnant cows are given little to no opportunity to find shelter (natural or artificial) once they are in the paddocks, and the standing surface, which is often uncovered, can quickly turn muddy and damp under wet weather (Calderón-Amor et al., 2021). Muddy paddock surfaces are detrimental to the welfare of dairy cows, particularly in wet and cold weather conditions (Neave et al., 2022). Studies have shown that prepartum dairy cows exposed to natural or simulated winter conditions spend more than 50% of their time inside an artificial shelter when allowed free access to it (Schütz et al., 2010; Cartes et al., 2021), likely because cows prefer dry lying surfaces (Tucker et al., 2021), which might be scarce during winter weather.

Although calving location preferences have been previously studied in outdoor areas (Edwards et al., 2020; Zobel et al., 2020), to our knowledge, no study has investigated whether, under natural winter conditions, cows would prefer to calve in an artificial shelter when given the opportunity. Thus, the main aim of our study was to assess the preference and use of an artificial shelter in dairy cows managed outdoors at calving during winter. We also evaluated whether this preference would be influenced by weather conditions (rain, temperature, or wind) or by the time of the day at calving. We hypothesized that cows would use the shelter to seek a dry surface as calving approaches.

This study was conducted from June to September 2020 (southern hemisphere winter) at the Austral Agricultural Experimental Station of the Universidad Austral de Chile in Valdivia, Chile. All procedures used were approved by the Universidad Austral de Chile Animal Ethics Committee under protocol #361/2020. Weather conditions such as precipitation (mm), air temperature (°C), relative humidity (%), and wind speed (m/s) were recorded daily from a weather station at the Institute of Agricultural Research of Chile (A720, ADCON Telemetry GmbH), located 1 km from the research location.

Three cohorts of 6 multiparous clinically healthy Holstein–Friesian cows calving in July (cohort 1), August (cohort 2), and September (cohort 3) were used. Body weight and BCS (5-point scale, Ferguson et al., 1994) were evaluated at the beginning of the prepartum period (3 wk before projected calving). Given the dimensions of the artificial shelters (area for 2 cows) and their limited availability, the sample size was restricted to 3 pairs of cows per cohort.

Before the study, from dry-off (approximately 8 wk before the expected calving date) until about 2 wk before the expected calving date, cows were kept in outdoor groups on paddocks and provided with pasture (mixture of grasses and legumes) and free access to drinking water. The stocking rate in these paddocks was approximately 20 to 25 cows/ha (400 to 500 m^2^/cow), but this was dynamic as cows entered and left the paddock depending on their expected calving date.

Cows were enrolled in the study at −16 ± 3 d (average ± SD) relative to their expected calving date and were paired based on BW, BCS, and parity. Mean parities (± SD) for cohorts 1, 2, and 3 were 2.8 ± 1.6, 2.5 ± 1.6, and 2.3 ± 1.3, respectively; BW were 614 ± 73 kg, 623 ± 102 kg, and 576 ± 51 kg, respectively; and BCS were 2.6 ± 0.2, 2.7 ± 0.4, and 2.5 ± 0.1, respectively. At enrollment, each cohort was moved on the same day from dry-off paddocks to the experimental paddock with access to an artificial shelter. Within 12 h after calving, the cow and her calf were removed from the paddock. After the first cow of the pair calved, the second cow remained alone in the paddock with access to the artificial shelter until she also calved. The calf was moved to the calf barn, and the cow was integrated into the lactating group.

Each experimental outdoor paddock measured 14 × 6 m (42 m^2^/cow) and had a bare soil surface without grass cover, reduced water infiltration, and high mud content. The amount of mud was controlled through a boot test used previously by our research group (Cartes et al., 2021) and adapted from Chen et al. (2017). This test evaluated how far mud came up while the researcher was standing on 2 spots inside of each outdoor paddock, on a scale of 3 points: 1 = dry soil (mud does not cover the boots); 2 = muddy soil (boots covered with mud below ankle level); and 3 = very muddy soil (mud-covered boots above ankle level). The amount of mud was maintained in a score of 2 or less by individually moving each group to an adjacent outdoor area.

An artificial shelter was located within each paddock. The artificial shelter consisted of a 6 × 3 m metal structure, with 3 sides covered with zinc sheets and a polycarbonate roof (see Cartes et al., 2021, for more details), placed on the back of each paddock with the opening toward the north. The ground surface of the shelter was covered with a thick layer of dry sawdust (approximately 15 cm deep), which was changed or added daily or whenever necessary to keep the lying area clean and dry. Manure was raked daily and removed from each shelter area (approximately at 0900 h). Each pair of cows interacted visually and auditorily with neighboring cows of the cohort, but their tactile contact was restricted due to electric fencing separating the paddocks.

Cows were fed twice a day, at approximately 0900 and 1500 h, and the ration was provided in 2 feed bins placed outside the artificial shelter. The ration was formulated following NRC (2001) guidelines and consisted of approximately 30 kg of grass silage per day on an as-fed basis (34% DM, 10% CP, 63% NDF, and 2.2 Mcal/kg DM) and approximately 3 kg of commercial concentrates per day on an as-fed basis (87% DM, 22.5% CP, 12.0% NDF, and 11.56 Mcal/kg DM). Also, each outdoor paddock was provided with fresh water ad libitum (600-L water trough).

The cows' use of artificial shelter and their behavior inside were monitored via continuous video recordings (Ezviz CS-CV310-A0–1B2WFR, California). The camera signal was captured through an NVR recorder (Ezviz Wi-Fi EZVIZ X5C, California). Two cameras were installed inside the indoor roof of each shelter. The area under observation was naturally lit during day hours (0800 to 1759 h), and infrared lighting was used for night recording (1800 to 0759 h). We considered that a cow was using the shelter when at least 3 legs were inside the shelter. Lying and sleeping behavior were analyzed when the cow was inside the shelter. Lying was defined as lying on sternal or lateral recumbence (or both) with the head lifted (Jensen, 2012). Sleeping was established as lying on sternal or lateral recumbence (or both) with the head resting on the ground or flank for at least 30 s (Ternman et al., 2014; Hunter et al., 2021). We also evaluated inside of the shelter, the number of lying bouts (i.e., frequency of transitions from lying to standing position), sleeping bouts (i.e., frequency of transition from sleeping to lying or standing position), and their respective average duration (i.e., calculated as the ratio of minutes lying or sleeping to the number of lying or sleeping bouts).

Calving time was defined as the time when the calf was fully expelled from the cow (Campler et al., 2015). Once calving time was established, each cow was continuously monitored in 2 periods: (1) the day before calving (baseline period), cows were monitored inside the artificial shelter from 48 to 24 h before calving time, and (2) day of calving, cows were monitored inside the artificial shelter the 24 h before calving time. One trained observer analyzed all behaviors.

Data were analyzed using R (version 4.0.3; https://www.r-project.org/) using the pair of cows as the experimental unit. Significant effects were defined as *P* < 0.05 and *P* < 0.001, and tendencies were considered at *P* < 0.10. Two cows were excluded from the analyses, one because she remained only 1 d with access to shelter and another due to failure in the video recording. Thus, 16 cows were used to analyze the preference and use of the artificial shelter. In a preliminary analysis of weather variables, we found that only precipitation was associated with behavior (*P* < 0.1). Thus, we categorized each day based on precipitation level (>1 mm/d = with rain, and ≤1 mm/d = without rain). We used the Fisher exact test to determine the association between calving location preferences (inside or outside artificial shelter) and the presence of rain at calving day (with or without) or time of the day when calving occurred (daytime = 0800 to 1759 h or nighttime = 1800 to 0759 h). We also used Linear Mixed Models, R package lme4 (Bates et al., 2015), and lmerTest (Kuznetsova et al., 2017) to determine the time (daily scale = min/d; hourly scale = min/h) of use of the shelter and expression of behaviors inside it (lying behavior = lying time, number lying bouts, duration lying bout; sleeping behavior = sleeping time, number sleeping bout, duration sleeping bout). First, we evaluated the behaviors on a daily scale by considering the period (day before calving and day of calving) and rain (with or without) as fixed effects and the cows' pair as a random effect. Second, to evaluate behavior on an hourly scale, we considered the period (day before calving and day of calving), and the time of day (day = 0800 to 1759 h and night = 1800 to 0759 h) as fixed effects and the cows' pair as a random effect. In all Linear Mixed Models, the normality of residuals was evaluated through graphic inspection, and we used the Akaike information criterion to select the appropriate covariance structure.

Our analyses revealed that most cows (94%, 15/16) calved inside the artificial shelter. We found no effect of rainfall on the use of the artificial shelter at calving (*P* > 0.1); 5 cows calved inside of the shelter on days with rain, 10 calved inside of the shelter on days without rain, and 1 calved outside of the shelter on a day without rain. A summary of the weather conditions recorded during the study period is provided in Table 1.

**Table 1:** Weather conditions (average ± SD; range) recorded during the day of calving and the day before calving

Item	Day before calving^1^	Day of calving^2^
Rainfall (mm)	4.3 ± 5.5; 0.0–16.5	1.9 ± 3.2; 0.0–10.3
Temperature (°C)	6.7 ± 1.3; 3.8–8.7	6.8 ± 2.8; 2.4–11.7
Humidity (%)	81 ± 5; 73–92	82 ± 10; 66–95
Wind velocity (m/s)	3.1 ± 1.3; 0.7–9.5	2.3 ± 2.2; 0.3–9.5

1 24 to 48 h before the calf was fully expelled.

2 24 h before the calf was fully expelled.

The preference to calve inside or outside the artificial shelter was not associated with the time of day (*P* > 0.1). Of the 16 cows, 9 calved inside the shelter during nighttime, 6 calved inside the shelter during the day, and 1 cow calved outside the shelter during the daytime.

Cows tended to spend more time inside the shelter on the calving day compared with the day before calving (*P* < 0.1) and, once inside the shelter, cows spent their time mainly lying (day before calving: 70%; day of calving: 62%; details in Table 2).

**Table 2:** Least squares means (± SEM) of daily use of artificial shelter and behavior inside the shelter for the day of calving and the day before calving in dairy cows (n = 16)

Item	Day before calving^1^	Day of calving^2^
Use of shelter (min/d)	895 ± 33	950 ± 32†
Behavior inside shelter		
Lying time (min/d)	627 ± 31	586 ± 41
Lying bouts (no./d)	24 ± 2	35 ± 2**
Lying bout duration (min)	25 ± 2	17 ± 2**
Sleeping time (min/d)	66 ± 5	45 ± 7*
Sleeping bouts (no./d)	12 ± 1	10 ± 1†
Sleeping bout duration (min)	5.7 ± 0.4	5.0 ± 0.5

1 24 to 48 h before the calf was fully expelled.

2 24 h before the calf was fully expelled.

†, *,** Indicate statistical difference in the same row, with †*P* < 0.1, *P < 0.05, and **P < 0.001.

Behaviors inside the shelter for the day of calving and the day before are presented in Table 2. Total daily lying time was similar on the day before calving and calving day (*P* > 0.1), but we observed that cow had a greater number of lying bouts (*P* < 0.001) and lower duration of lying bouts (*P* < 0.001) on the day of calving compared with the day before calving. Cows spent less time sleeping (*P* < 0.05) and tended to have fewer sleeping bouts (*P* < 0.1) on the calving day compared with the day before calving. However, the duration of the sleeping bouts did not differ between the day before calving and calving day (*P* > 0.1).

The time of the day influenced shelter use; cows spent more time using the shelter during nighttime than during the daytime (*P* < 0.05; Figure 1A). On the day of calving and the day before, cows spent more time lying (*P* < 0.05; Figure 1B), had more lying bouts (day before calving: 1.4 ± 0.1 bouts/h vs. 0.7 ± 0.1 bouts/h; day of calving: 1.8 ± 0.1 bouts/h vs. 1.0 ± 0.1 bouts/h; *P* < 0.05), spent more time sleeping (*P* < 0.05; Figure 1C), had more sleeping bouts (*P* < 0.05; Figure 1D), and had longer sleeping bout duration (day before calving: 6.6 ± 0.4 min vs. 4.3 ± 0.7 min; day of calving: 5.5 ± 0.5 min vs. 3.8 ± 0.8 min; *P* < 0.05) during the nighttime than during the daytime. The time of day did not influence the lying bout duration (day before calving: 33 ± 2 min vs. 32 ± 3 min; day of calving: 26 ± 2 min vs. 25 ± 3 min; *P* > 0.1).

**Figure 1:**
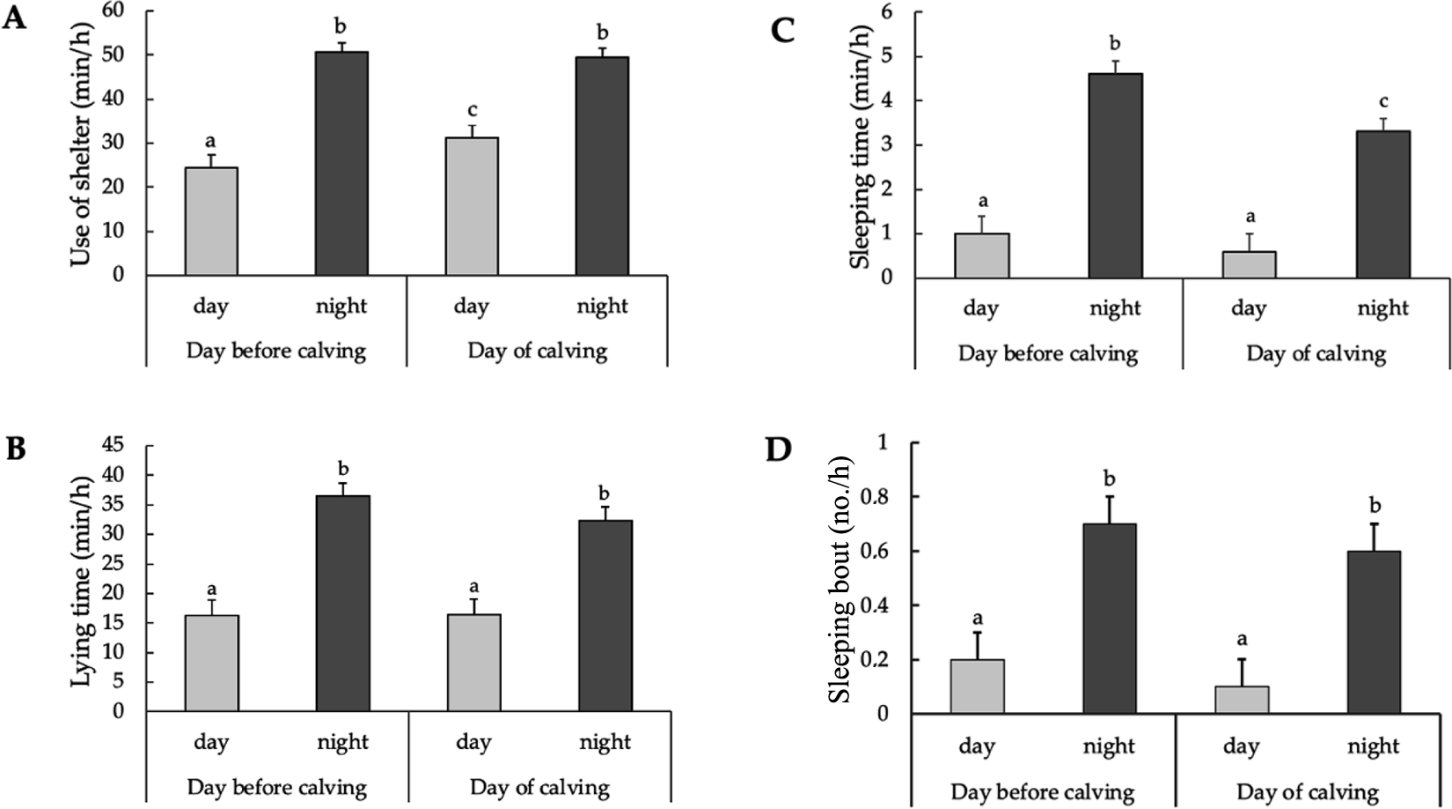
Least squares means (± SEM) of (A) use of artificial shelter (min/h), (B) lying time (min/h), (C) sleeping time (min/h), and (D) sleeping bouts (no./h) according to time of day (day = 0800 to 1759 h, and night = 1800 to 0759 h) for the day of calving and the day before calving in dairy cows (n = 16). Different letters (a–c) show differences in mean values (*P* < 0.05).

Our findings showed dairy cows preferred using an artificial shelter during the periparturient period when managed outdoors on sacrifice paddocks during winter, suggesting that access to this resource was important for them. The quality of the lying surface inside the shelter may explain this preference; the shelter's roof protected against precipitation and, thus, maintained a drier space for the cows to lie down. Our decision to bed the surfaces with a thick layer of dry sawdust was based on previous work showing the importance of having access to comfortable lying surfaces during winter when cows are managed outdoors (Schütz and Cox, 2014; Schütz et al., 2015, 2019). It should be noted that cows could have chosen to use a similar dry-bedded surface in the paddock. Further studies are needed to evaluate whether the use of the shelter at calving is influenced by other factors in addition to the dry bedding, such as the presence of herd mates, social hierarchy, or different stocking densities.

Cows' preferences for calving inside the artificial shelter and the time they spent using it were not affected by the presence of rainfall, low temperature, humidity, or windy weather. These results are similar to those reported in cows exposed to artificial (Schütz et al., 2010) and natural winter conditions (Cartes et al., 2021) in temperate climates. Schütz et al. (2010) suggested that prepartum cows may be driven to seek shelter for reasons other than protection from weather. One possible explanation may include seeking isolation, which could be particularly important for cows nearing calving (Proudfoot et al., 2014a). Under natural conditions, cows isolate from their herd to seek an undisturbed calving process (Rørvang et al., 2018) and imprint bond with their offspring (Orihuela et al., 2021). Further research is needed to understand the consequences of providing shelter on calving behavior and its impact on the calving process or risk of dystocia.

Cow preference for calving inside or outside the artificial shelter was not associated with the time of the day. Our result supports those reported by Edwards et al. (2020) and Zobel et al. (2020), who did not find an effect of time of the day on the preference for calving location in cows managed outdoors. In contrast, Proudfoot et al. (2014b) found that cows in an indoor system preferred calving in a hiding area during the daytime, but only when they were housed alone. Proudfoot et al. (2014b) speculated that environmental factors in indoor systems, such as changes in light and constant human activity, could explain their results.

Regardless of the day of study (the day before calving or the day of calving), once inside the artificial shelter, the cows spent most of their time lying down. Campler et al. (2019) suggested that the motivation for lying increases as both BW and body size increase closer to calving, making it essential to adopt calving management practices that promote the expression of this behavior. It is also well known that dairy cattle in off-pasture situations prefer and spend more time lying on soft, well-bedded, and dry surfaces (see review by Tucker et al., 2021), suggesting that cows entered the shelters when motivated to lie down. On the other hand, cows increased the frequency of lying bouts on the day of calving compared with the day before calving, which has been described as the result of “normal” discomfort associated with the proximity to calve (Huzzey et al., 2005; Saint-Dizier and Chastant-Maillard, 2015).

On average, cows slept 50 min per day inside of the artificial shelter. Ternman et al. (2019) reported that cows spent around 1 h daily during late pregnancy in REM sleep; thus, we suppose that our cows only slept inside the artificial shelter. There was a decrease in sleeping time on the calving day, likely associated with the cow's alertness during the calving process. However, there were no relevant changes in daily number of sleeping bouts or their duration. It should be noted that sleep behavior estimation is more precise for measuring the number of sleeping bouts than quantifying sleeping time (Ternman et al., 2014). Undoubtedly, cow sleep behavior is an area that needs more understanding.

Our results are similar to those from previous studies, showing that cows exposed to winter conditions under temperate climates seek and use an artificial shelter more during nighttime than daytime (Van Laer et al., 2015; Cartes et al., 2021). This may be due to cow preference to lie down at night, as shown by others for pastured dairy cattle (O'Connell et al., 1989; Hendriks et al., 2019); on average, we found that cows spent more time lying and sleeping during the nighttime than during the daytime. Additional contributing factors to our results may include prioritizing behaviors such as feeding or grazing over the motivation to seek and use shelter during the day (Van Laer et al., 2015).

The findings of this study have to be seen in light of some limitations. As we tested the calving place preference under natural winter conditions, we had problems with recording video cameras on windy and rainy days, with the consequent reduction to 2 d for the evaluation of time use of the artificial shelter. Another limitation of our study was the small sample size used. This was related to the need to form pairs of cows homogeneous in BW, BCS, and parity, and expected calving dates within each cohort, which led to several cows being excluded from this study due to having no partner. In addition, we had a limited number of artificial shelters, which were built for research purposes (they were not part of the farm infrastructure). It is also important to mention that our research did not explore whether the shelter was simultaneously used by 1 or 2 cows within the pair, potentially affecting shelter usage. Understanding how more complex social herd dynamics and competition may influence shelters at a calving time in dairy cows exposed to muddy and wet conditions requires further investigation with larger groups of animals.

In conclusion, dairy cows managed in small groups in wet and muddy sacrifice paddocks preferred to use an artificial shelter for calving. The preference for the artificial shelter to calve was not influenced by weather conditions or by the time of day. Both on the day before calving and the day of calving, the cows spent most of the time using the artificial shelter, and once inside it, they spent the time mainly lying down. In addition, regardless of the day, the cows spent more time using the artificial shelter in the nighttime than in the daytime. Thus, our results show the importance of providing artificial shelter with dry bedding as a resource for protection from muddy conditions to dairy cows managed outdoors precalving and at the time of calving.
